# RETRACTION: Antibacterial, Anti‐Inflammatory, Antioxidant, and Antiproliferative Properties of Essential Oils from Hairy and Normal Roots of *Leonurus sibiricus* L. and Their Chemical Composition

**DOI:** 10.1155/omcl/9843537

**Published:** 2026-07-30

**Authors:** Oxidative Medicine and Cellular Longevity

RETRACTION: P. Sitarek, P. Rijo, C. Garcia, et al., “Antibacterial, Anti‐Inflammatory, Antioxidant, and Antiproliferative Properties of Essential Oils from Hairy and Normal Roots of *Leonurus sibiricus* L. and Their Chemical Composition,” *Oxidative Medicine and Cellular Longevity*, 2017, 7384061, https://doi.org/10.1155/2017/7384061.

The above article, published online on 16 January 2017 in Wiley Online Library (wileyonlinelibrary.com), has been retracted retracted by agreement between the Journal Editor‐in‐Chief, Jeannette Vasquez Vivar; and John Wiley & Sons Ltd. The retraction has been agreed due to concerns that have been raised with Figure 3b, also reported on PubPeer [1]. Specifically, there are multiple instances of unexpectedly similar bands shown in Figure 3b, reported under both the same and different conditions.

Moreover, multiple bands in Figure 3b also appear highly similar to ones in Figure 3b of the author’s previous work [2], assembled in apparently random order in a different context. Lastly, the sixth IL‐6 band also appears highly similar to ones in Figure 2a in another of the author’s previous publications [3], assembled in apparently random order in a different context.

The authors responded to the concerns; however, due to the time elapsed since the study, they could not provide the original images for this figure. After assessing the response to the concerns, we conclude that neither the results nor the conclusions can be considered reliable, and the article is therefore retracted.

The authors disagree with the retraction.
